# Supplementary material for: Deep transfer learning-based hologram classification for molecular diagnostics
Source: Sci Rep. 2018 Nov 19;8:17003. doi: 10.1038/s41598-018-35274-x (PMC6242900; doi:10.1038/s41598-018-35274-x)
Supplement: Supplementary file 1 — Supplementary Information [file 41598_2018_35274_MOESM1_ESM.pdf]

Supplementary Information

Deep transfer learning-based hologram classification for molecular diagnostics

Sung-Jin Kim[1*], Chuangqi Wang[1*], Bing Zhao[2], Hyungsoon Im[4,5], Jouha Min[4,5], Hee June Choi[1], Joseph Tadros[1], Nu Ri Choi[1], Cesar M. Castro[4], Ralph Weissleder[4,5,6], Hakho Lee[4,5], Kwonmoo Lee[1,3]

[1]Department of Biomedical Engineering, Worcester Polytechnic Institute, Worcester, Massachusetts

[2]Department of Computer Science, Worcester Polytechnic Institute, Worcester, Massachusetts

[3]Department of Electrical and Computer Engineering, Worcester Polytechnic Institute, Worcester, Massachusetts

[4]Center for Systems Biology, Massachusetts General Hospital, Boston, Massachusetts

[5]Department of Radiology, Massachusetts General Hospital, Boston, Massachusetts

[6]Department of Systems Biology, Harvard Medical School, Boston, Massachusetts

[*]These authors equally contributed to this work.

Correspondence

Kwonmoo Lee (klee@wpi.edu), Hakho Lee (hlee@mgh.harvard.edu)

Classification Type	Holograms			Object Images		
	PCA-MLP	VGG19-PCA-MLP	P-values	PCA-MLP	VGG19-PCA-MLP	P-values
N/P	0.795 (0.020)	0.902 (0.015)	6.62×10^{-08}	0.768 (0.017)	0.934 (0.009)	6.57×10^{-08}
N/P+BG	0.791 (0.013)	0.904 (0.009)	6.70×10^{-08}	0.764 (0.013)	0.923 (0.011)	6.64×10^{-08}
N_B	0.528 (0.022)	0.755 (0.027)	6.73×10^{-08}	0.534 (0.019)	0.820 (0.021)	6.70×10^{-08}
N_B +BG	0.658 (0.010)	0.832 (0.011)	6.65×10^{-08}	0.643 (0.014)	0.862 (0.009)	6.63×10^{-08}

Supplementary Table 1. Accuracies of VGG19-PCA-MLP and PCA-MLP classifiers. The values within parentheses are the standard deviations. The p-values testing the difference between PCA-MLP and VGG19-PCA-MLP were obtained by Wilcoxon rank sum test.

Classification Type	Holograms			Object Images		
	PCA-MLP	VGG19-PCA-MLP	P-values	PCA-MLP	VGG19-PCA-MLP	P-values
N/P	0.861 (0.031)	0.925 (0.017)	1.02×10^{-07}	0.820 (0.027)	0.947 (0.015)	6.52×10^{-08}
N/P+BG	0.782 (0.029)	0.902 (0.022)	6.64×10^{-08}	0.749 (0.036)	0.926 (0.021)	6.56×10^{-08}

Supplementary Table 2. Sensitivities of VGG19-PCA-MLP and PCA-MLP classifiers. The values within parentheses are the standard deviations. The p-values testing the difference between PCA-MLP and VGG19-PCA-MLP were obtained by Wilcoxon rank sum test.

Classification Type	Holograms			Object Images		
	PCA-MLP	VGG19-PCA-MLP	P-values	PCA-MLP	VGG19-PCA-MLP	P-values
N/P	0.695 (0.051)	0.867 (0.034)	8.26×10^{-08}	0.690 (0.043)	0.915 (0.017)	6.30×10^{-08}
N/P+BG	0.865 (0.016)	0.936 (0.014)	6.69×10^{-08}	0.850 (0.023)	0.951 (0.011)	6.50×10^{-08}

Supplementary Table 3. Specificities of VGG19-PCA-MLP and PCA-MLP classifiers. The values within parentheses are the standard deviations. The p-values testing the difference between PCA-MLP and VGG19-PCA-MLP were obtained by Wilcoxon rank sum test.

Classification Type	Holograms			Object Images		
	PCA-MLP	VGG19-PCA-MLP	P-values	PCA-MLP	VGG19-PCA-MLP	P-values
N/P	0.565 (0.043)	0.795 (0.032)	6.79×10^{-08}	0.514 (0.035)	0.863 (0.019)	6.79×10^{-08}
N/P+BG	0.672 (0.019)	0.849 (0.014)	6.79×10^{-08}	0.628 (0.021)	0.879 (0.017)	6.79×10^{-08}
N_B	0.392 (0.028)	0.687 (0.034)	6.79×10^{-08}	0.403 (0.024)	0.770 (0.027)	6.79×10^{-08}
N_B +BG	0.522 (0.014)	0.766 (0.015)	6.79×10^{-08}	0.500 (0.019)	0.808 (0.013)	6.79×10^{-08}

Supplementary Table 4. Cohen's Kappa of VGG19-PCA-MLP and PCA-MLP classifiers. The values within parentheses are the standard deviations. The p-values testing the difference between PCA-MLP and VGG19-PCA-MLP were obtained by Wilcoxon rank sum test (All p-values are the same since they have the same rank order distribution and there is no overlap of the data distribution of VGG19-PCA-MLP and PCA-MLP)

Classification Type	Holograms			Objects		
	PCA-MLP	VGG19-PCA-MLP	P-values	PCA-MLP	VGG19-PCA-MLP	P-values
N/P	0.251 (0.040)	0.528 (0.052)	6.786×10^{-08}	0.204 (0.030)	0.643 (0.036)	6.796×10^{-08}
N/P+BG	0.403 (0.023)	0.664 (0.021)	6.796×10^{-08}	0.358 (0.025)	0.704 (0.033)	6.796×10^{-08}
N_B	0.204 (0.020)	0.487 (0.045)	6.796×10^{-08}	0.190 (0.018)	0.599 (0.034)	6.796×10^{-08}
N_B +BG	0.314 (0.015)	0.609 (0.018)	6.796×10^{-08}	0.275 (0.021)	0.664 (0.017)	6.796×10^{-08}

Supplementary Table 5. RCI values of VGG19-PCA-MLP and PCA-MLP classifiers. The values within parentheses are the standard deviations. The p-values testing the difference between PCA-MLP and VGG19-PCA-MLP were obtained by Wilcoxon rank sum test (All p-values are the same since they have the same rank order distribution and there is no overlap of the data distribution of VGG19-PCA-MLP and PCA-MLP).

P-values	MLP VS SVM	MLP VS RF	SVM VS RF
Accuracy	6.532×10^{-08}	6.588×10^{-08}	4.356×10^{-06}
Specificity	1.054×10^{-04}	6.560×10^{-08}	6.504×10^{-08}
Sensitivity	9.197×10^{-08}	6.279×10^{-07}	6.188×10^{-08}

Supplementary Table 6. P-values of the hypothesis testing of the differences in the performance measures among MLP, SVM, and RF in N/P classification. Wilcoxon rank-sum test was used

P-values	MLP VS SVM	MLP VS RF	SVM VS RF
Accuracy	1.094×10^{-05}	6.710×10^{-08}	7.184×10^{-08}
Kappa	1.415×10^{-05}	6.796×10^{-08}	6.796×10^{-08}
RCI	2.925×10^{-05}	6.796×10^{-08}	6.796×10^{-08}

Supplementary Table 7. P-values of the hypothesis testing of the differences in the performance measures among MLP, SVM, and RF in N_B classification. Wilcoxon rank-sum test was used.

	Accuracy	Kappa	RCI
P-values	0.3571	0.3507	1

Supplementary Table 8. P-values of the hypothesis testing of the differences in the performance measures between VGG19-PCA-MLP and CNN in N_B classification. Wilcoxon rank-sum test was used.
